# Insulin-like growth factors and liver cancer risk in male smokers

**DOI:** 10.1038/sj.bjc.6605842

**Published:** 2010-08-17

**Authors:** J M Major, R Z Stolzenberg-Solomon, M N Pollak, K Snyder, J Virtamo, D Albanes

**Affiliations:** 1 Division of Cancer Epidemiology and Genetics, National Cancer Institute, NIH, Bethesda, MD 20852, USA; 2 Department of Oncology, Jewish General Hospital and McGill University, Montreal, Quebec, Canada H3T 1E2; 3 Information Management Services Inc., Silver Spring, MD 20904, USA; 4 Department of Chronic Disease Prevention, National Institute for Health and Welfare, Helsinki 00300, Finland

**Keywords:** insulin-like growth factor, IGF-I, incidence, men

## Abstract

**Background:**

The liver is the primary source of circulating insulin-like growth factor (IGF)-I, yet the relation between IGFs and liver cancer is uncertain.

**Methods:**

In a case–cohort study within a cohort of 29 133 male smokers we examined associations of serum IGF-I and IGF binding protein (IGFBP)-3 with liver cancer (50 cases).

**Results:**

Nonlinear associations between liver cancer and IGF-I and IGFBP-3 were observed (*P*=0.04 and *P*<0.01, respectively), strongest association at lowest levels (odds ratio (OR)=0.2, 95% confidence interval (CI)=0.1–0.7 for 80 *vs* 30 ng ml^−1^ of IGF-I; OR=0.2, 95% CI=0.1–0.6 for 1400 *vs* 700 ng ml^−1^ of IGFBP-3).

**Conclusions:**

Low IGF-I and IGFBP-3 levels in male smokers are associated with increased risk of liver cancer.

Liver cancer is the fifth most common cancer worldwide (Bosch *et al*, 2004), occurs more commonly in men, and has a high fatality among affected patients. Although populations of developing countries have the highest risk with the primary cause being hepatitis B virus (HBV) infection, a trend of increasing incidence has been observed in developed countries (McGlynn *et al*, 2001). Hepatocellular carcinoma (HCC) accounts for over 85% of cases, but underlying mechanisms of hepatocarcinogenesis are not completely understood and may vary across risk factors (Scharf and Braulke, 2003).

The liver is the primary source of circulating insulin-like growth factor(IGF)-I, a peptide hormone of which levels peak at puberty and decrease thereafter (Juul, 2003; Pavelic *et al*, 2007). IGF-I has been implicated in the regulation of cancer cells through effects on proliferation, differentiation, and apoptosis (Dunn *et al*, 1997; Pollak, 2000; Moschos and Mantzoros, 2002; Sachdev and Yee, 2007). Pituitary secretion of growth hormone stimulates hepatic IGF-I production, and more than 75% of circulating IGF-I is bound and regulated by IGF binding protein (IGFBP)-3 (Ferry *et al*, 1999). Although previous studies showed an association between high serum concentrations of IGF-I and risk of prostate, colorectal, and breast cancers (Rowlands *et al*, 2009; Chan *et al*, 1998, 2002; Ma *et al*, 1999; Yu *et al*, 2002; Renehan *et al*, 2004), subsequent studies have found low IGF-I levels to be associated with an increased risk for brain and kidney cancer (Lonn *et al*, 2007; Major *et al*, 2010). Clinical studies suggest a possible inverse IGF-liver function association, although reverse causality, with low IGF-I levels being a consequence of liver damage, remains a possibility (Wu *et al*, 1988; Buzzelli *et al*, 1993). Several studies reported the reduced IGF levels to be independent of liver function and progression of disease, providing support for a potential causal connection (Stuver *et al*, 2000; Mazziotti *et al*, 2002; Mattera *et al*, 2003; Elsammak *et al*, 2006). However, these studies involved patients who already had cirrhosis or hepatitis C virus (HCV)-related liver disease.

Unlike above investigations, the *α*-Tocopherol, *β*-Carotene Cancer Prevention (ATBC) Study has a prospective design and involved a large population of men who, although being smokers, did not have liver cirrhosis, chronic alcohol abuse, or cancer at enrolment. In addition, the ATBC Study was conducted in Finland, a developed country with an extremely low prevalence of viral infections of the liver (e.g., HBV, HCV). We conducted a case–cohort study to assess the independent roles of IGF-I and IGFBP-3 in liver cancer aetiology in a population without preexisting diseases that are known risk factors.

## Materials and methods

The ATBC Study was a randomised, placebo-controlled trial that evaluated the effect of supplementation with *α*-tocopherol, *β*-carotene or both on the incidence of cancer (The ATBC Cancer Prevention Study Group, 1994). In 1985–88, 29 133 eligible men in southwestern Finland, aged 50–69 years, who smoked at least five cigarettes daily, were randomised to an intervention group. Exclusion criteria included previous diagnosis of cancer (excluding non-melanoma skin cancer), liver cirrhosis, chronic alcoholism, or other medical conditions that might limit long-term participation. All study participants gave written informed consent before participation; the study was approved by the Institutional Review Boards of the National Public Health Institute in Finland and the US National Cancer Institute.

At study entry, participants completed an investigator-administered baseline questionnaire that included data on demographics, smoking habits, and medical history. Trained study staff measured height and weight at baseline using standard methods. Diet, including alcohol intake, was assessed with a validated self-administered food frequency questionnaire. Fasting serum samples were collected from participants at their baseline visit and stored at −70°C until analyzed. IGF assays were performed in the laboratory of Dr Pollak (McGill University, Montreal, QC, Canada). Total serum IGF-I and IGFBP-3 were measured by enzyme-linked immunosorbent assays after separation of IGFs from IGFBPs by acid-ethanol extraction (Diagnostic Systems Laboratory, Inc., Houston, TX, USA). The inter-batch assay coefficient of variation (CV) was 4.6 and 6.2% for IGF-I and IGFBP-3, respectively; the intra-batch assay CV was 5.2 and 4.2%, respectively.

All participants in the current study were alive and without evidence of cancer during the first 5 years of follow-up. Incident cases of liver cancer were defined based on the International Classification of Diseases 9; codes 155.0, 155.1, and 155.2. Cases were identified through the Finnish Cancer Registry, which provides almost 100% case ascertainment (Korhonen *et al*, 2002). Medical records for cases with a major cancer site were reviewed centrally by two study physicians to confirm diagnoses (The ATBC Cancer Prevention Study Group, 1994). Cancers metastasised from other organ sites are not included in our complement of cases. A subcohort group of 400 men was randomly selected from the full ATBC Study population, representing the comparison group. Follow-up for the current study included time from randomisation through 31 December 1997, date of diagnosis, or date of death (whichever occurred first).

### Statistical analysis

Exposures of interest included baseline serum IGF-I, IGFBP-3, and the IGF-I/IGFBP-3 molar ratio. Potential confounders included age, anthropometric measures, lifestyle factors, and medical history. IGF-I/IGFBP-3 molar ratio was calculated as a measure of bioavailability using the following conversion: 1 ng ml^−1^ IGF-I =0.130 nmol l^−1^ and 1 ng ml^−1^ IGFBP-3 =0.036 nmol l^−1^. Body mass index was calculated as weight in kilograms divided by squared height in metres (kg m^−2^).

General linear models adjusted for age were used to estimate least square means and standard errors to help identify potential confounders. Group comparisons for continuous variables were performed using analysis of covariance; categorical variables were compared using logistic regression. In addition, correlations between IGF-I, IGFBP-3, and covariates adjusting for age were calculated using Pearson's partial correlations in the comparison group.

Given the small number of cases (*n*=50), IGF measures were analyzed as continuous predictors of liver cancer to maximise study power to identify associations. Logistic regression models were used to determine the appropriate functional form (e.g., linear or quadratic; Woodward, 2005). Accordingly, models for IGF-I and IGFBP-3 included a linear and a quadratic term to account for the curvilinear relation with liver cancer risk (Hosmer and Lemeshow, 2000; Jewell, 2004). IGF-I/IGFBP-3 molar ratio was modelled as a continuous linear variable. Odds ratios (ORs) and corresponding 95% confidence intervals were estimated for age- and multivariable-adjusted models. Consistent with the interpretation of a continuous variable, we report the ORs for 1-unit increments (e.g., 1-s.d.). As for nonlinear associations the estimated risks will vary across the IGF-axis, a single ‘overall’ OR cannot be calculated across the whole range of IGF concentrations; therefore, we report the ORs for four consecutive 1-s.d. increments.

All models included a continuous variable for age. Other variables were included in the final model if they were associated with both the exposure of interest and disease, changed the OR by at least 10%, or had a *P-*value ⩽0.20 in the full model. The multivariable analyses did not include more covariates than appropriate for the given number of cases (Peduzzi *et al*, 1996). Possible effect modifications by body weight, cigarettes smoked, and alcohol intake were examined by evaluating interaction terms in the models; no significant interactions were observed.

## Results

Among the 50 incident liver cancer cases, 88% were adenocarcinomas. Age-adjusted characteristics at baseline are shown in Table 1. On average, cases were older (mean age at randomisation=60 years), smoked more cigarettes daily, were less physically active during leisure time, and had lower serum cholesterol. Mean serum levels of IGF-I and IGFBP-3 were significantly lower in cases than in the comparison group, after adjusting for age: 115 *vs* 146 ng ml^−1^ and 2018 *vs* 2388 ng ml^−1^, respectively (*P*<0.01). Intervention group assignment was not associated with liver cancer (*P*=0.28; data not shown).

Table 2 shows age and age-adjusted correlations of baseline participant characteristics with IGF-I and IGFBP-3. As shown, inversely correlated with serum IGF-I were age (*P*=0.03), alcohol intake (*P*=0.04), and having diabetes (*P*=0.03), whereas body weight and IGFBP-3 were positively associated with IGF-I levels (*P*<0.01), with IGFBP-3 and IGF-I having a moderately high correlation of *r*=0.69.

Age-adjusted logistic regression analyses show a significant nonlinear association between liver cancer risk and serum IGF-I and IGFBP-3 (*P*<0.05 and *P*<0.01, respectively; Table 3), suggesting some curvature to the relationship between risk and IGF levels such that the ORs vary across the range of IGF values. Hence, ORs and 95% confidence intervals (CIs) are reported for consecutive 1-s.d. intervals of 50 ng ml^−1^ for IGF-I (700 ng ml^−1^ for IGFBP-3). In the present study, risk of liver cancer was 78% lower for men with levels of 79 ng ml^−1^ compared with 30 ng ml^−1^, 66% lower for 129 ng ml^−1^ compared with 80 ng ml^−1^, and 47% lower at 179 ng ml^−1^ compared with 130 ng ml^−1^, after adjustment for age. The ratio of cases to comparison group in the lowest range was 11 : 31 (35%). These results suggest that men with low levels of IGF had greater risk. Study findings were not markedly different after further adjusting for cigarettes smoked and physical activity. IGFBP-3 levels did not alter the association between serum IGF-I and risk of developing liver cancer. Similarly, risk was statistically significant for the lowest IGFBP-3 levels; IGF-I levels did not alter the risk-IGFBP-3 association (Table 3).

Although there was no evidence of a nonlinear relation (*P*=0.38 for quadratic term) between the IGF-I/IGFBP-3 molar ratio and risk, there was a suggested linear association such that for every 0.05-unit decrease in molar ratio, the odds of developing liver cancer increased by a factor of 1.35 (=1.0/0.74), after adjustment for age, daily cigarettes, and physical activity (OR=0.74; 95% CI=0.53–1.01), but this was not significant (*P*=0.06).

## Discussion

This is the first population-based study to prospectively examine the association of circulating IGF-I and IGFBP-3 levels with development of liver cancer, doing so within a cohort of older middle-aged men who, although smokers, did not have a history of alcoholism, liver cirrhosis, or previous cancer, and who resided in a country that has low prevalence of HBV and HCV infections. We observed significant nonlinear associations between IGF-I and IGFBP-3 and liver cancer risk, with the strongest association seen in the lowest IGF range. These associations were not altered markedly after adjusting for potential confounders. Interpretation of results should be made with caution, however, given the small number of cases.

Strengths of this investigation include the prospective study design of the ATBC Study, in which IGF-I and IGFBP-3 were measured from blood samples collected at the baseline visit, preceding the development of liver cancer. The possibility that IGF levels were influenced by subclinical liver cancer was minimised by selecting participants who were alive and without clinical evidence of cancer during the first 5 years of cohort follow-up. A limitation of this study was the inability to directly adjust for aflatoxin, HBV, and HCV, established risk factors for liver cancer (London and McGlynn, 2006), even though the prevalence of HBV and HCV infections in Finland is low; that is, <0.001 and 0.4%, respectively (Iivonen *et al*, 2005; Shepard *et al*, 2005, 2006).

Our findings are consistent with those from the only other study of liver cancer and IGF-1, in which patients with HCC were found to have significantly lower IGF-I levels than age- and gender-matched controls after adjusting for prothrombin time and serum albumin levels, indicators of the degree of liver damage (Stuver *et al*, 2000). Given the complexity of the IGF system and the paucity of population-based studies examining the IGF-liver cancer association, the pathophysiological mechanisms potentially involved will remain for subsequent investigation to elucidate. In conclusion, findings from this study suggest that IGF-I and IGFBP-3 may have independent roles in development of liver cancer. Confirmation of our results in other large, prospective population-based studies is needed, including examination of associations in women and nonsmokers.

## Figures and Tables

**Table 1:** Age and age-adjusted baseline characteristics of men, ATBC Study

**Characteristic**	**Cases (*n*=50)**	**Comparison group (*n*=400)**	***P-*value**
Age at randomisation (years)	60 (0.7)	56 (0.2)	<0.01
Height (cm)	174.0 (0.9)	173.7 (0.3)	0.79
Weight (kg)	82.1 (1.9)	80.3 (0.7)	0.38
BMI (kg m^−2^)	27.0 (0.6)	26.6 (0.2)	0.45
Serum total cholesterol (mmol l^−1^)	6.0 (0.2)	6.3 (0.1)	0.04
Alcohol intake (g per day)	22.3 (3.6)	19.2 (1.2)	0.43
			
*Smoking history*			
No. of cigarettes per day	23.7 (1.2)	20.3 (0.4)	<0.01
No. of years smoked	36.8 (1.0)	35.3 (0.3)	0.16
*Physical activity – leisure, n* (%)			0.01
Light	31 (65.4)	172 (42.6)	<0.01
Moderate	18 (33.4)	204 (51.3)	0.02
Heavy	1 (1.2)	24 (6.1)	0.16
			
*Medical history*			
Diabetes, *n* (%)	5 (9.4)	18 (4.6)	0.16
Gallstones, *n* (%)	2 (2.9)	21 (5.4)	0.47
Insulin-like growth factor-I (ng ml^−1^)	115.2 (7.3)	146.3 (2.5)	<0.01
Insulin-like growth factor BP-3 (ng ml^−1^)	2017.8 (92.5)	2387.9 (31.9)	<0.01
IGF-I/IGFBP-3 molar ratio	0.21 (0.01)	0.22 (0.003)	0.07

Abbreviations: ATBC=*α*-Tocopherol, *β*-Carotene Cancer Prevention; BMI=body mass index; IGF-I=insulin-like growth factor-I; IGFBP-3=IGF binding protein-3

Means (s.e.) are reported unless otherwise indicated.

*P-*values from generalised linear models.

**Table 2:** Age and age-adjusted partial correlations

	**IGF-I**		**IGFBP-3**	
**Characteristic**	***r***	***P-*value**	***r***	***P-*value**
Age (years)	−0.11	0.03	−0.21	<0.01
Height (cm)	0.19	<0.01	0.17	<0.01
Weight (kg)	0.17	<0.01	0.25	<0.01
BMI (kg m^−2^)	0.10	0.05	0.19	<0.01
Serum total cholesterol (mmol l^−1^)	0.07	0.16	0.21	<0.01
Alcohol (g per day)	−0.10	0.04	−0.06	0.22
No. of cigarettes per day	−0.06	0.22	−0.05	0.32
No. of years smoked	−0.04	0.48	−0.09	0.08
Leisure physical activity (moderate+light)	0.09	0.06	0.12	0.02
Diabetes (yes/no)^a^	−0.11	0.03	−0.05	0.36
History of gallstones (yes/no)	−0.01	0.85	0.07	0.17
IGFBP-3 (ng ml^−1^)	0.69	<0.01	—	—

Abbreviations: BMI=body mass index; IGF-I=insulin-like growth factor-I; IGFBP-3=IGF binding protein-3.

*r* indicates Pearson's partial correlations among noncases (*n*=400).

a Diabetes=history of diabetes and/or fasting glucose ⩾126 mg dl^−1^.

**Table 3:** Associations between serum IGF measures and development of liver cancer

		**Model 1**			**Model 2**		
**Characteristic**	**Cases**	**OR**	**95% CI**	***P-*value**	**OR**	**95% CI**	***P-*value**
*IGF-I (per 50 ng m* ^−1^ *l)*				0.025			0.036
30–79	11	0.22	0.10–0.48		0.24	0.09–0.67	
80–129	24	0.34	0.21–0.54		0.38	0.19–0.75	
130–179	11	0.53	0.38–0.74		0.59	0.36–0.98	
180+	4	0.83	0.48–1.44		0.92	0.49–1.72	
							
*IGFBP-3 (per 700 ng ml*^−1^)				0.002			0.004
700–1399	14	0.14	0.06–0.35		0.23	0.08–0.64	
1400–2099	21	0.30	0.18–0.49		0.45	0.23–0.88	
2100–2799	9	0.63	0.45–0.87		0.91	0.55–1.49	
2800+	6	1.30	0.69–2.46		1.82	0.90–3.66	
							
*IGF-I/IGFBP-3 molar ratio (per 0.05 units)*							
	50	0.74	0.54–1.01	0.057	0.73	0.53–1.01	0.060

Abbreviations: CI=confidence interval; IGF-I=insulin-like growth factor-I; IGFBP-3=IGF binding protein-3; OR=odds ratio.

The association of IGF was modelled as a continuous (rather than categorical) predictor of liver cancer. IGF-I and IGFBP-3 were centred to prevent collinearity with the quadratic term.

1-s.d. increase in IGF-I=50 ng ml^−1^; 1-s.d. increase in IGFBP-3=700 ng ml^−1^; *P-*value based on the Wald test for quadratic term (IGF-I; IGFBP-3) and linear term (molar ratio).

Model 1: age-adjusted.

Model 2: adjusted for age, cigarettes per day, leisure physical activity, and IGFBP-3; adjusted for age, cigarettes per day, leisure physical activity, and IGF-I; adjusted for age, cigarettes per day, and leisure physical activity.
